# The TANDEM trial: protocol for the process evaluation of a randomised trial of a complex intervention for anxiety and/or depression in people living with chronic obstructive pulmonary disease (COPD)

**DOI:** 10.1186/s13063-021-05460-w

**Published:** 2021-07-26

**Authors:** Moira Kelly, Liz Steed, Ratna Sohanpal, Hilary Pinnock, Amy Barradell, Clarisse Dibao-Dina, Kristie-Marie Mammoliti, Vari Wileman, Vickie Rowland, Sian Newton, Anna Moore, Stephanie Taylor

**Affiliations:** 1 Centre for Primary Care and Mental Health, Institute of Population Health Sciences, Barts and The London School of Medicine and Dentistry, Yvonne Carter Building, 58, Turner Street, London, E1 2AB UK; 2 Allergy and Respiratory Research Group, Usher Institute of Population Health Sciences and Informatics, Doorway 3, Medical School, Teviot Place, Edinburgh, EH8 9AG UK; 3 Department of Respiratory Sciences, College of Life Sciences, NIHR Leicester Biomedical Research Centre- Respiratory Glenfield Hospital, University of Leicester, Groby Road, Leicester, LE3 9QP UK; 4 Université de Tours, Université de Nantes, INSERM, SPHERE U1246, 10 Boulevard Tonnellé, B.P. 3223, 37044 Tours, cedex 1 France; 5 Birmingham Clinical Trials Unit and WHO Collaborating Centre for Global Women’s Health Research, University of Birmingham, Birmingham, B15 2TT UK; 6 Department of Health & Social Sciences, University of the West of England, Frenchay Campus, Coldharbour Lane, Bristol, BS16 1QY UK; 7 The Education Academy, Barts Health NHS Trust, Royal London Hospital, Whitechapel Road, London, E1 1FR UK

**Keywords:** Process evaluation, Implementation, Complex intervention, Chronic obstructive pulmonary disease (COPD), Depression, Anxiety, Cognitive behavioural therapy (CBT)

## Abstract

**Background:**

TANDEM is a randomised controlled trial of a complex healthcare intervention to improve the psychological and physical health of people living with chronic obstructive pulmonary disease (COPD) and anxiety and/or depression. Based on health psychology theory set out in a logic model, respiratory health professionals were recruited and trained to deliver a cognitive behavioural approach intervention (The TANDEM intervention) under the supervision of senior cognitive behavioural practitioners. Here, we describe the protocol for the process evaluation commissioned alongside the trial. A realist approach that includes attention to describing contexts and mechanisms has been adopted.

**Methods:**

We set up a multi-disciplinary team to develop and deliver the process evaluation. The mixed-methods design incorporates quantitative process data; monitoring of intervention fidelity; qualitative interviews with patients, carers, health professionals (facilitators) and clinical supervisors about their perspectives on acceptability of the intervention; and exploration with all stakeholders (including management/policy-makers) on future implementation. Normalisation process theory (NPT) will inform data collection and interpretation with a focus on implementation. Quantitative process data will be analysed descriptively. Qualitative interview data will be analysed before the trial outcomes are known using analytic induction and constant comparison to develop themes. Findings from the different elements will be reported separately and then integrated.

**Conclusion:**

Detailed description and analysis of study processes in a research trial such as TANDEM enables research teams to describe study contexts and mechanisms and to examine the relationship with outcomes. In this way, learning from the trial goes beyond the randomised control trial (RCT) model where effectiveness is prioritised and makes it possible to explore issues arising for post-trial study implementation.

**Trial registration:**

ISRCTN ISRCTN59537391. Registered on 20 March 2017. Trial protocol version 6.0, 22 April 2018.

Process evaluation protocol version 4.0, 1 November 2020.

## Background

Chronic obstructive pulmonary disease (COPD) is a complex long-term condition (LTC) associated with considerable morbidity and mortality. It has a global prevalence of 11.7% in adults aged over 30 years [1]. It is considered to be a neglected disease [2] despite being the third leading cause of death worldwide, the fifth biggest cause of death in the UK [3], and the ninth cause of disability-adjusted life years [4]. Pulmonary rehabilitation (PR) is an effective therapeutic approach for people with COPD [5, 6]. However, both referrals from health professionals and attendance at PR courses by patients are sub-optimal [7]. COPD often co-exists with other LTCs, including anxiety (10–50%) [8, 9], and depression (30%) [8], which affect hospital admissions, quality of life and self-efficacy [10, 11]. The evidence for optimal approaches to managing psychological comorbidities in COPD is not conclusive, and there are few evidence-based care pathways for anxiety and depression in people with COPD [12]. Psychological therapies such as cognitive behavioural therapy (CBT) are proposed as potential treatments for patients living with LTCs such as COPD with co-morbid anxiety and/or depression [13–15]. Research suggests that the beneficial effects of psychological treatment for anxiety and depression on its own in COPD are limited and therefore studies have reported linking psychological treatment with physical activity, lifestyle and self-management support interventions to improve patient outcomes [16]. Despite much discussion about the need to provide patient-centred care for people with LTCs that integrates mental and physical health, health professionals often find addressing psychological aspects challenging [17, 18].

This paper presents the protocol for a process evaluation being undertaken in parallel to a trial evaluating a tailored intervention using a cognitive behavioural approach (CBA) incorporating self-management skills which precedes, links with, and optimises the benefits of currently offered PR, in patients with COPD and depression and/or anxiety. The intervention incorporates training and supervision of respiratory healthcare professionals to deliver five to eight individual CBA sessions to patients whilst awaiting PR. The multi-centre, pragmatic, randomised controlled trial (RCT), including an internal pilot, will assess whether receiving the intervention (TANDEM) prior to routine PR improves anxiety and/or depression in people with moderate to very severe COPD (GOLD criteria [19]) and mild to moderate anxiety and/or depression. The trial will also assess if the intervention improves attendance and completion of PR. A parallel economic evaluation will be undertaken. The main trial protocol has been published [20].

### The TANDEM randomised controlled trial

We hypothesise that the TANDEM intervention will improve anxiety and/or depression and consequently encourage uptake and completion of PR.

TANDEM trial objectives are:

1. To undertake a RCT of the TANDEM intervention to examine the effectiveness of this intervention on clinical outcomes compared to usual care (i.e. the offer of PR alone).
2. To examine the effect of the TANDEM intervention (which is directed at patients) on their carers (where appropriate).
3. To determine the cost-effectiveness of the TANDEM intervention from a National Health Service (NHS) and personal social services perspective.
4. To conduct a process evaluation to inform the implementation of the TANDEM intervention if the trial is positive, or assist interpretation of the findings if it is negative.

**Fig. 1:**
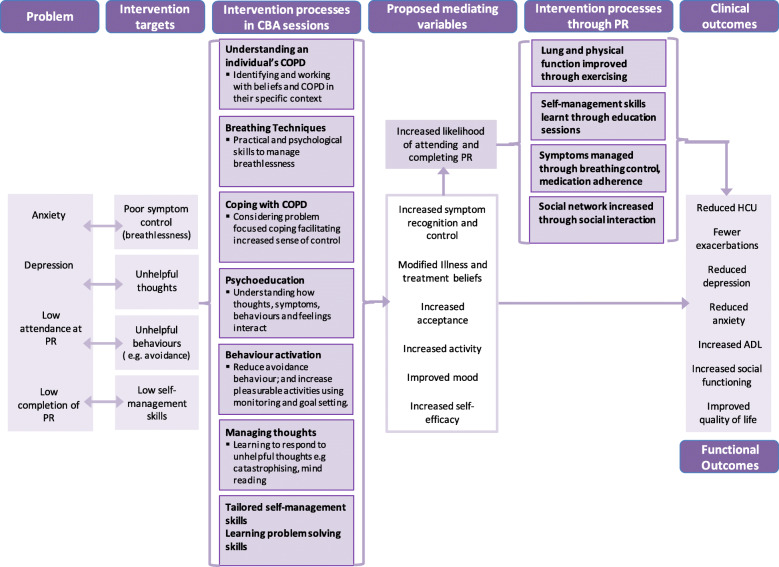
Logic model for TANDEM intervention

TANDEM is a complex intervention meaning that it is composed of multiple interacting components [21]. The multi-level intervention integrates both CBA training of health professionals and their delivery of CBA to patients. We have developed an intervention logic model [22] which maps out a causal chain for the problem under investigation and identifies the intervention mechanisms, which aspects will be changed, and how (see Fig. 1). The model uses an intervention based on Beck’s cognitive behaviour therapy model for managing anxiety and depression [23], as well as self-management training informed by Bandura’s social cognitive theory [24, 25], and Leventhal’s self-regulation theory [26]. The logic model is based on the premise that the way in which individuals with COPD think about their illness has an influence on how they behave and feel. Change targeted at cognitive, behavioural or symptom level will improve emotional outcomes and self-management making it more likely that people will attend PR [27].

Respiratory healthcare professionals (including physiotherapists, nurses, and occupational therapists), termed TANDEM facilitators, have been recruited, trained and are supervised to deliver tailored, manualised sessions, that draw on the principles of cognitive behavioural therapy, to NHS patients living with COPD and co-morbid mild to moderate anxiety and/or depression. We describe this as a cognitive behavioural approach (CBA) intervention [27]. TANDEM facilitators deliver five to eight (with nine maximum weekly CBA sessions to allow for a missed session, e.g. due to illness), each lasting 40–60 min, in a location preferred by the patient, and if the patient decides to attend PR the facilitators provide further telephone support sessions before starting PR and continuing to two weeks following completion of PR. Facilitators receive telephone clinical supervision by a trained cognitive behavioural practitioner. Patients (and their carers) have been recruited from 12 participating National Health Service (NHS) Trusts partnered with five NHS Clinical Commissioning Groups (CCGs). Facilitators have been recruited among the participating study sites and from neighbouring NHS organisations across England.

### Process evaluation

Process evaluation is viewed as an essential element in trials of complex interventions [28]. It is used to assess fidelity, the quality of implementation, causal mechanisms, and to identify contextual factors associated with variation in outcomes [21]. A process evaluation can provide additional information for interpreting trial results and making decisions about whether the intervention is likely to work in a wider context [29]. If the intervention is found not to work, detailed description of the processes involved in delivery can help to explain whether this was due to problems with the intervention or failure to implement the intervention as planned [30].

The value of concurrently evaluating implementation alongside effectiveness in a hybrid design has been proposed [31], and this is typically done in a process evaluation. TANDEM is an example of a hybrid type 1 design which involves testing a clinical intervention whilst gathering information on delivery during the effectiveness trial and/or on its potential for implementation in the real-world [31]. The TANDEM process evaluation was set up after initial development work and formative evaluation, involving pre-pilot and pilot studies, which is reported by Steed et al. [27].

## Process evaluation methods

### Underpinning theory and considerations for the process evaluation

**Fig. 2:**
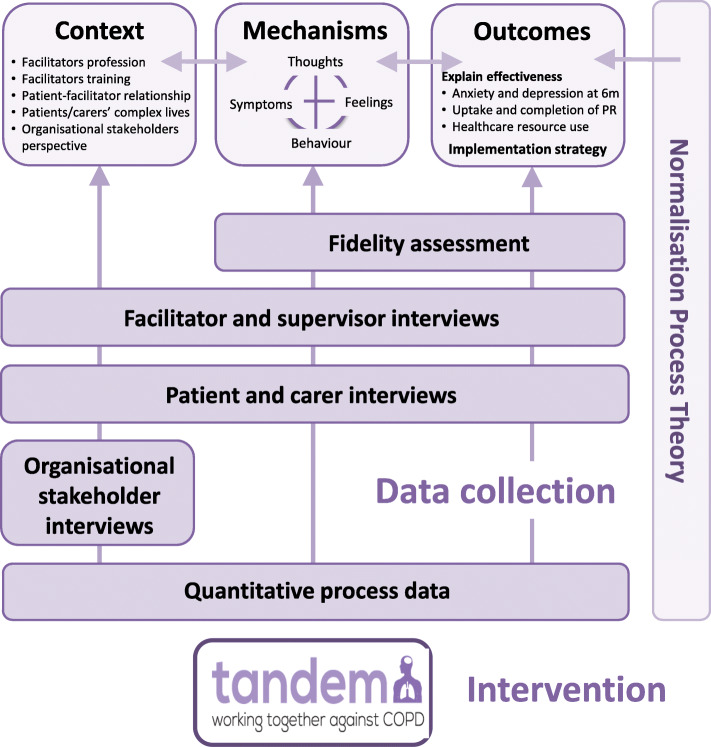
TANDEM process evaluation conceptual framework

Complex interventions are now presented as interactions of theory, context and implementation, rather than a set of mechanisms of change across multiple domains [32]. Our process evaluation, in line with MRC guidance [33], is broadly informed by the principles of realist evaluation [34, 35]. Realist evaluation is an evaluation framework that attends to what it is about an intervention that works, for whom it works, and in what circumstances. It examines phenomena in relation to how context, mechanism and outcome are configured as patterns [34]. All Interventions are underpinned by theoretical assumptions about change and this may involve more than one theory, especially in complex interventions. We held a workshop to discuss and agree conceptual models, creating a multi-dimensional framework that aims to make the different elements visible in order to maximise the field of vision in terms of breadth and depth, in turn enabling fuller engagement with the complex systems in which complex interventions are undertaken [36]. The conceptual framework for the TANDEM process evaluation can be seen in Fig. 2.

Theory informs the TANDEM process evaluation in three main ways:

- ➢ Realist evaluation principles of context, mechanism, outcome underpin the overall approach to our process evaluation design (understanding how the intervention works)
- ➢ Programme theory using psychological theory is set out as a logic model linking causal mechanisms (theory of change)
- ➢ Normalisation process theory (NPT) will be used to consider implementation

In addition the process evaluation, similarly to the TANDEM intervention and study, is underpinned by a strong patient centred ethos [37].

**Fig. 3:**
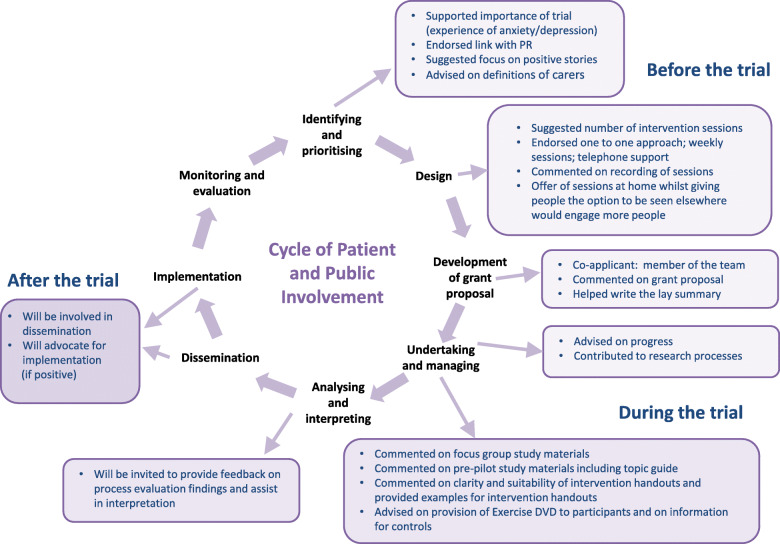
Patient and public involvement in the TANDEM study

An intervention can be considered as an event in a dynamic complex system [38], where viewing the intervention dynamically affects how the reach and effectiveness can be improved. There has been an emphasis on engaging from the early stages of study development with those who would receive the intervention (patients and carers) and those who would deliver it (health care professionals) in order to optimise participant engagement with the study tools and the relevance of the intervention to real-world implementation. Patient and public involvement in TANDEM, including the process evaluation, can be seen in Fig. 3. The trial participants (patients, carers, health care professionals) are treated as active agents, who interact with the intervention mechanisms within a specified set of system conditions and shape the systems in which they are nested.

When considering outcomes, we aim to understand how participants interact with the intervention mechanisms so that change is generated. For example, people living with COPD often experience multimorbidity and have complex lives that affect their experience of care [39, 40], meaning that it can be difficult to isolate the impact of the COPD. It is also not possible to separate the facilitators’ experiences of delivering the intervention from the contexts in which they work including their professional identity. We need therefore to describe contexts, how contexts influence system change and how the intervention modifies the context. Potentially salient contextual influences on effectiveness and implementation that we have identified for the TANDEM trial are as follows: facilitators’ professional practice; facilitator training; patient-facilitator relationship; facilitator supervision; patient (and carer where relevant) complex lives, policy-level priorities and organisational stakeholders’ perspectives on the delivery context. These will be explored using qualitative methods which are described below.

Explication of conceptual frameworks is key to guiding the post-trial implementation process [41]; however, although formal and informal theory in implementation studies is viewed as important, it is often poorly described and under-recognised [42]. NPT challenges researchers to consider how their intervention might become embedded or ‘normalised’ (or not) into routine practice [41, 43]. In NPT, the work involved in implementation is investigated by focusing on: how people make sense of the intervention (coherence); how people participate in the intervention (cognitive participation); how they act collectively (collective action); and how they reflect and monitor what is happening (reflexive monitoring) [44].

### Aims and objectives

The overall aim of our process evaluation is to describe and understand the processes by which the trial is conducted (specifically including fidelity and acceptability to recipients and professionals) and to consider the effect of these on the outcomes of the study. The process evaluation will also inform the implementation of the TANDEM intervention if the trial outcome is positive, or assist in the interpretation of findings if it is negative. The objectives address: acceptability, fidelity and implementation.

Specific objectives are:

1. To assess the acceptability of the intervention to patients and carers, including consideration of: content (in session, home practice); therapeutic alliance; and practicalities (location, timing).
2. To assess the acceptability of the intervention to facilitators and supervisors delivering the intervention.
  - Patient-facing CBA sessions including content, structure, logistics, telephone support and integration of components.
  - Facilitator training including content, logistics, supervision, perceived confidence to deliver the CBA sessions.
  - Management of workload.
  - Supervisors’ training and workload.
3. To monitor the delivery of the intervention through assessment of fidelity.
  - Was the facilitator training delivered as intended with respect to professional competence?
  - Were the CBA sessions delivered as intended with respect to adherence and competency?
4. To consider the feasibility of implementing the intervention with respect to:
  - The recruitment, training and retention of facilitators and organisation of clinical supervision.
  - Rates of completed delivery of at least two CBA sessions (pre-determined estimate of minimal clinically important “dose” of intervention) per patient.
  - Numbers of patients seen by facilitators and numbers of sessions delivered to patients and reasons for intervention non-attendance/ no delivery of sessions.
  - Intervention drop-out or disruption to delivery and reason for drop out or disruption.
5. To explore the experiences and perspectives of patients, carers, facilitators, and supervisors regarding the intervention and post-trial implementation.
  - What are patients, carers, facilitators and supervisors’ experiences of the intervention and what are their views about its potential impact on health and quality of care?
  - What are the barriers and facilitators to implementation and how do these vary according to context and/or other factors?
  - Were there any unexpected consequences?
6. To explore the views of organisational stakeholders regarding post-trial implementation of the intervention.

- What are the barriers and facilitators to implementation and how do these vary according to context and/or other factors?
- What resources and partnerships are necessary for implementation?
- To understand whether adaptations to the intervention are necessary depending on the clinical context in which it takes place e.g. if the intervention is delivered through primary care, secondary care or solely via PR services.

### Research design and methods

This is a mixed-methods study using quantitative and qualitative methods. We are collecting quantitative and qualitative data from the intervention arm of the trial (and quantitative data from the control arm). The process evaluation lead (MK) is not involved in the design or delivery of the trial itself. RS, AB, KM and VR are involved in trial recruitment and data collection. The different types of data collected are described below and are separated for practical purposes but will be integrated and drawn upon in complementary ways in order to address the process evaluation aims and objectives. For example, qualitative data from patient and facilitator interviews may be used to triangulate with findings from the fidelity analysis.

### Quantitative process data

Quantitative data are being collected throughout the multi-level intervention in order to understand whether delivering the intervention was feasible, assess the workload required for delivery, and how this may have varied from the trial protocol. Structured data will be presented descriptively and will be collected via:

- Facilitator recruitment, training attendance and training completion rates.
- Facilitator retention and caseloads of facilitators.
- Data logs on uptake, attendance, cancelled/re-scheduled appointments and completion of CBA sessions by patients.
- CBA content logs at the end of each face-to face patient session and analysis of audio-recordings of sessions.
- Subsequent attendance at PR (including number of PR sessions attended)
- Telephone support sessions by facilitator.

### Qualitative data collection

Qualitative research can explore complex phenomena and areas less amenable to quantitative research [45] and aid understanding of the processes involved in both intervention and evaluation. Central to our process evaluation is collecting qualitative interview data that will allow us to describe, review and interpret the trial processes, including mechanisms and contexts, by gaining in-depth understanding of the perspectives of research participants. The qualitative data are being collected via three sub-studies: experiences and views of patients and carers on receiving the intervention (RS, AB, KM), experiences and views of facilitators and supervisors on training and delivering the CBA sessions (SN, AM) and views of organisational stakeholders (clinical commissioners, GPs, PR specialists, nurses, psychologists) on the implementation context (VW, VR). To reduce the risk of bias, interviewers are not interviewing participants they have recruited to the study. Details of the qualitative data collection and outline topic guides can be seen in Table 1.

**Table 1:** Qualitative data collection methods

	Sample	Main issues addressed by topic guide
Patients	5 participants who completed face to face TANDEM sessions (after 6-month follow-up assessment) 5 participants who dropped out of TANDEM (< 4 CBA sessions) (after 6-month follow-up assessment) 5 participants who completed TANDEM CBA sessions and PR programme (after 6/12-month follow-up assessment) 5 participants who completed TANDEM but dropped out or did not attend PR (after 12-month follow-up assessment)	• Current experience of COPD/breathlessness • Experience of being in the TANDEM study • Relationship and working with the TANDEM facilitator • Experience of attending PR • Suggested improvements to the TANDEM experience • Perspectives on receiving TANDEM as part of routine care
Carers of intervention participants	5 (after 6-month follow-up assessment)	• Relationship with patient/role • Understanding of TANDEM • Perspectives on CBA sessions • Experience of care role in the study • Any observed improvements in patient’s condition/quality of life
Facilitators	Up to 14 All facilitators to be invited, but aim for range of professional group and number of patients seen	• Training sessions • CBA sessions with patients • Supervision • Professional identity • Perspectives on post-trial implementation
Clinical supervisors	Up to 4 All invited	• Training • Clinical supervision sessions • Logistics of organising supervision sessions • Providing clinical supervision for professions who do not usually receive it
Organisational stakeholders	Up to 20 interviews Range of organisational context and roles	• Organisation and role • Issues faced in delivering and improving COPD services • Perspectives on the value of PR for people with COPD • Understanding of TANDEM • Views on the TANDEM approach to care • Perceived differences with current care approaches for COPD • Perspectives on post-trial implementation of TANDEM • Facilitators and barriers for implementation • Commissioning

Interviews are being undertaken by telephone or in person depending upon the preference of the research participant and the COVID-19 pandemic restrictions. Topic guides for the interviews with patients, carers, facilitators and supervisors are based on those used in the developmental and pilot phases. The topic guide for the organisational stakeholders draws on issues arising in discussions within the trial team. With a view to gaining an understanding of issues for implementation, topic guides have also been informed by the key NPT constructs of coherence, cognitive participation, collective action and reflexive monitoring [44]. Through this user-centred approach, we expect to gain insight into contextual influences and the facilitators and barriers to post-trial implementation. The NPT concepts will support our analysis and help to identify implementation challenges and areas where further evaluation is needed. The topic guides provide structure and aim to elicit participant experiences and perspectives in the context of their lives and work roles. Open questions are used to explore issues in the terms of participants and to allow for unexpected issues to emerge.

Sampling frames have been drawn up based on a purposive sampling approach aiming for maximum variation [46] to gain a full range of views. Sampling is being reviewed during data collection and data analysis so that if unexpected themes emerge additional perspectives can be sought. If saturation is reached data collection will stop [47].

### Data analysis

Quantitative process data will be analysed and presented using simple descriptive statistics (e.g. counts and proportions). Qualitative data will initially be analysed thematically using an inductive approach and constant comparison [48]. Group discussion in research teams improves the rigour and quality of qualitative research [49], so our analysis will be a reflexive, iterative process involving review and multidisciplinary discussion. NVivo 12 software will be used to assist in the organisation and analysis of the data. A thematic narrative will be constructed for each sub-study.

Our approach is to undertake theoretically informative research in addition to theoretically informed research [50]. Further analysis will therefore be undertaken, with second level themes identified and explored in the light of relevant theory and research literature [50, 51]. Data will also be interpreted drawing upon NPT resources such as the NPT toolkit [52] to assist interpretation of the data regarding post-trial implementation.

### Fidelity assessment

Assessment of intervention fidelity follows the American Behaviour Change Consortium framework [53] and aims to find out whether the intervention was delivered as intended and the quantity (dose) of intervention implemented. Should the trial outcome be negative fidelity assessment makes it possible to understand whether it is the intervention that is ineffective or if delivery failed [30]. Complex interventions usually undergo some tailoring/adaptation when delivered in different contexts. Capturing what is delivered in practice with reference to the theory of the intervention can enable evaluators to understand the core elements and where more flexibility may be allowed. Analysis focuses on what was delivered and how it was delivered, including training and support, communication and logistics, and how these structures interact with the implementer facilitators’ attitudes and circumstances to shape the intervention.

Fidelity will be assessed at two levels:

- The delivery of the 3-day facilitator training was recorded. Preliminary assessment of facilitators’ skills was made on days 2 and 3 of the training where possible using the IAPT low intensity practitioner assessment manual (based on Blackburn’s Cognitive Therapy Scale [54] by at least two trainers.
- Audio-recordings of all intervention sessions will be made. A random 25% sample of recorded sessions across all 25 CBA interventions, and a smaller sample of 10% of CBA entire interventions will be coded with respect to facilitator adherence to the manual and competence in trained skills. The fidelity assessment framework is reported in detail by Steed et al (Steed L, Wileman V, Sohanpal R, Kelly M, Pinnock H, Taylor S. Assessing fidelity in the TANDEM study: Strategies for enhancement and a protocol for assessment, in preparation).

### Integrating results of analysis

The different evaluation elements will be reported separately and then integrated before the trial results are known. The main trial findings will be analysed independently of the process evaluation findings. Once both analyses are complete the analyses will be combined.

We aim to produce a high-quality, integrated evaluation of the trial processes informed by a clear conceptual framework. We will ensure rigour across our analyses by being transparent, maintaining a clear account of the procedures used in an audit trail. We are addressing validity by providing evidence to support our interpretations and providing context. We are also undertaking a comprehensive analysis of each data set. Workshops with patients and carers will be set up, depending on their preference and convenience, to consider interpretation of the trial findings in the light of the process evaluation analysis. Workshops will also be held with facilitators to discuss findings from the process evaluation and explore issues around implementation, including CBA training and engaging patients.

## Conclusion

Providing detailed description and analysis of study processes in a research trial such as TANDEM can enable research teams to examine and interpret how study contexts and mechanisms contribute to outcomes. In this way, learning from the trial goes beyond the RCT model where effectiveness is prioritised. It also makes it possible to explore issues arising for post-trial study implementation.

As with many process evaluations, in designing our study, we have had to think strategically and prioritise areas to cover due to limited resources. We have produced an integrated, organic design (rather than a linear framework) that aims to optimise the usefulness of the TANDEM trial findings. We will be in a position to capture ‘messy realities’ in our process evaluation [32] that will provide insights into the complex and pragmatic issues emerging and how to respond to them. This includes consideration of context at an early stage to optimise implementation [41]. A specific—and unexpected—issue arising during the TANDEM trial is the impact of the COVID-19 pandemic on patients (many at high risk), stretched healthcare professionals and diverted research capacity.

## Data Availability

Not applicable.
